# Prediction of mortality in pneumonia patients with connective tissue disease treated with glucocorticoids or/and immunosuppressants by machine learning

**DOI:** 10.3389/fimmu.2023.1192369

**Published:** 2023-05-25

**Authors:** Dongdong Li, Liting Ding, Jiao Luo, Qiu-Gen Li

**Affiliations:** ^1^Medical College of Nanchang University, Nanchang, Jiangxi, China; ^2^Department of Pulmonary and Critical Care Medicine, Jiangxi Provincial People’s Hospital, The First Affiliated Hospital of Nanchang Medical College, Nanchang, Jiangxi, China; ^3^Department of Rheumatology and Clinical Immunology, Jiangxi Provincial People’s Hospital, The First Affiliated Hospital of Nanchang Medical College, Nanchang, Jiangxi, China; ^4^JXHC Key Laboratory of Rheumatology and Immunology, Jiangxi Provincial People’s Hospital, The First Affiliated Hospital of Nanchang Medical College, Nanchang, Jiangxi, China

**Keywords:** connective tissue disease, glucocorticoids, pneumonia, machine learning, prognosis

## Abstract

**Objectives:**

The assessment of accurate mortality risk is essential for managing pneumonia patients with connective tissue disease (CTD) treated with glucocorticoids or/and immunosuppressants. This study aimed to construct a nomogram for predicting 90-day mortality in pneumonia patients using machine learning.

**Methods:**

Data were obtained from the DRYAD database. Pneumonia patients with CTD were screened. The samples were randomly divided into a training cohort (70%) and a validation cohort (30%). A univariate Cox regression analysis was used to screen for prognostic variables in the training cohort. Prognostic variables were entered into the least absolute shrinkage and selection operator (Lasso) and a random survival forest (RSF) analysis was used to screen important prognostic variables. The overlapping prognostic variables of the two algorithms were entered into the stepwise Cox regression analysis to screen the main prognostic variables and construct a model. Model predictive power was assessed using the C-index, the calibration curve, and the clinical subgroup analysis (age, gender, interstitial lung disease, diabetes mellitus). The clinical benefits of the model were assessed using a decision curve analysis (DCA). Similarly, the C-index was calculated and the calibration curve was plotted to verify the model stability in the validation cohort.

**Results:**

A total of 368 pneumonia patients with CTD (training cohort: 247; validation cohort: 121) treated with glucocorticoids or/and immunosuppressants were included. The univariate Cox regression analysis obtained 19 prognostic variables. Lasso and RSF algorithms obtained eight overlapping variables. The overlapping variables were entered into a stepwise Cox regression to obtain five variables (fever, cyanosis, blood urea nitrogen, ganciclovir treatment, and anti-pseudomonas treatment), and a prognostic model was constructed based on the five variables. The C-index of the construction nomogram of the training cohort was 0.808. The calibration curve, DCA results, and clinical subgroup analysis showed that the model also had good predictive power. Similarly, the C-index of the model in the validation cohort was 0.762 and the calibration curve had good predictive value.

**Conclusion:**

In this study, the nomogram developed performed well in predicting the 90-day risk of death in pneumonia patients with CTD treated with glucocorticoids or/and immunosuppressants.

## Background

Connective tissue disease (CTD) is a series of autoimmune diseases that often leads to collagen deposition, tissue damage, and eventual target organ failure (1). Glucocorticoids and immunosuppressants are the main therapeutic agents to mitigate the progression of CTD (rheumatoid arthritis (2), systemic lupus erythematosus (3), systemic sclerosis (4)). However, patients receiving glucocorticoids or/and immunosuppressants often suffer pulmonary infections, which are one of the causes of death in patients (5, 6). The overall annual incidence of lower respiratory tract infections in rheumatoid arthritis patients taking oral steroids and disease-modifying antirheumatic drugs is 2.3% and the mortality rate is 22.5% (7). The prognostic factors for pneumonia in patients with CTD receiving glucocorticoids or/and immunosuppressants are currently unknown. Therefore, it is crucial to assess the risk of death from pulmonary infections in patients with CTD receiving glucocorticoids or/and immunosuppressants.

The severity assessment of patients with pneumonia is one of the most important predictors of patient prognosis. Several scoring methods exist for the assessment of the severity of community-acquired pneumonia (CAP) (8), which include the pneumonia severity index (PSI) and CURB-65 (confusion, uremia, elevated respiratory rate, hypotension, and aged 65 years or older). The PSI has been shown to have high discriminatory power for CAP mortality (9). However, the PSI includes 20 different variables and has limited application in a busy medical practice. The CURB-65 includes only five variables and is easy to use, and its ability to discriminate CAP mortality is almost comparable to that of the PSI. However, the PSI and the CURB-65 are heavily influenced by age (10). In addition, the poor performance of the CURB-65 and the PSI in predicting pneumonia mortality in immunocompromised populations has been reported (11). Therefore, there is a need to construct new prognostic models for assessing the prognosis of pneumonia patients with CTD who are using glucocorticoids or/and immunosuppressants.

Machine learning is a branch of artificial intelligence that uses algorithms to identify relationships in data (12) and has been used for different purposes (classification and prognosis) in various areas of biomedicine, such as transcriptomics, radiomics, and drug response predictions (13). Chen et al. used machine learning to screen 14 variables associated with pneumonia after liver transplantation (14). Gao et al.’s early warning system based on machine learning can accurately predict the risk of death from COVID-19 (15). Machine learning (random forest) can predict mortality in pneumonia patients more accurately than the existing CURB-65 and can help determine whether a patient should be admitted to the intensive care unit (16). However, no studies have been reported on the construction of prognostic models based on machine learning for patients with CTD pneumonia who are using glucocorticoids or/and immunosuppressive drugs.

We constructed a comprehensive predictive nomogram by using machine learning algorithms, clinical features, and laboratory test indicators. In addition, we validated the prognostic performance of the model using an internal validation cohort.

## Methods

### Study population

**Figure 1:**
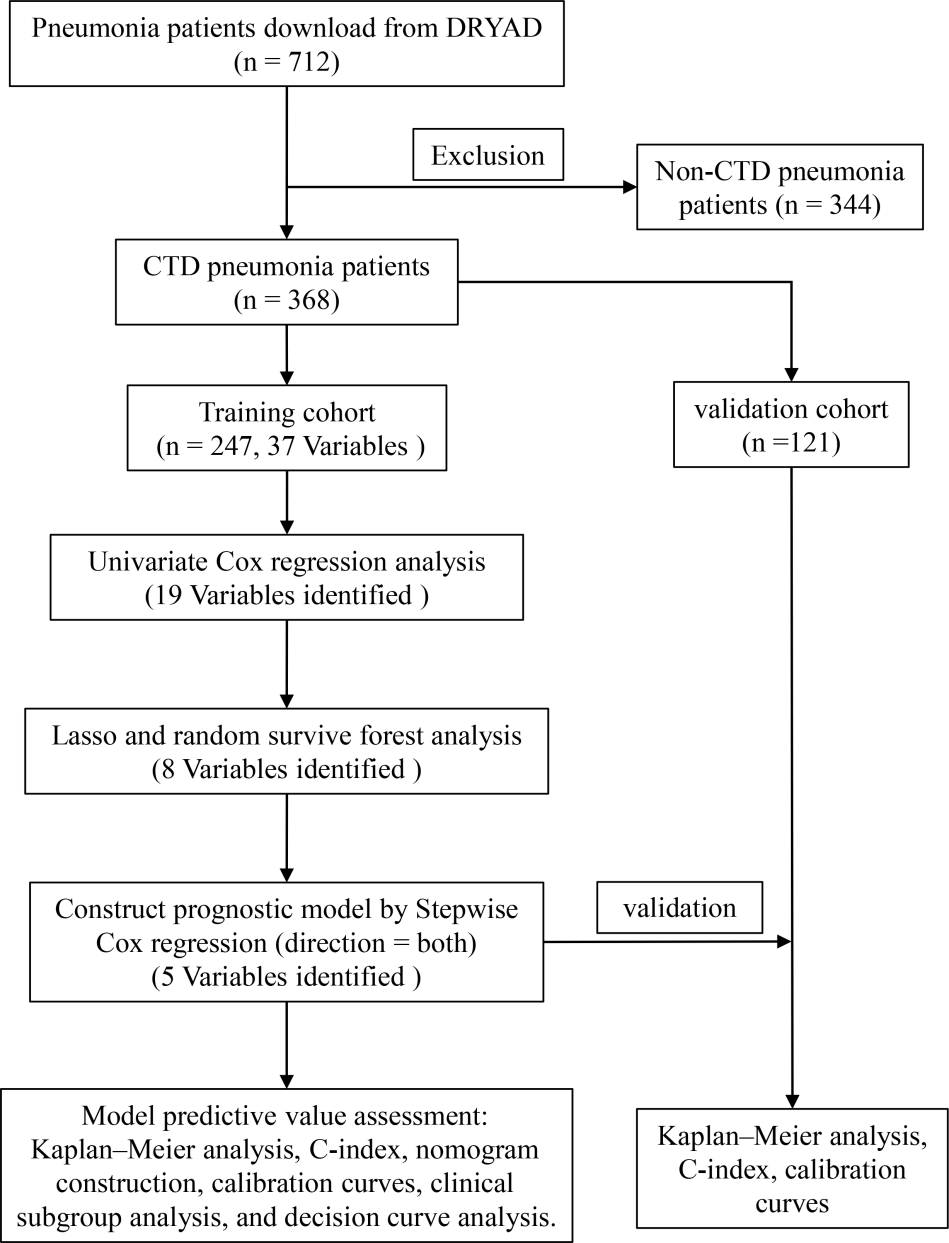
Flow chart for this study.

The study was based on a previously reported cohort that recruited patients with pneumonia who were hospitalized in six secondary and tertiary academic hospitals in China between January 1, 2013 and December 31, 2017 (17). The diagnosis of pneumonia was based on the American Thoracic Society guidelines and guidelines for infectious diseases (6, 18). The study inclusion criteria were as follows: (1) oral or intravenous glucocorticoid therapy, (2) diagnosis of pneumonia during admission and hospitalization, and (3) age 18 years or older. Exclusion criteria were as follows: (1) diagnosis of non-infectious lung disease, including lung cancer, interstitial lung disease without infection, pulmonary embolism, or heart failure, and (2) inability to provide informed consent. The Ethics Committee of China–Japan Friendship Hospital approved the retrospective study and organized centralized collaboration and approval of all participating institutions. The flow chart is shown in **Figure 1**.

### Data download and processing

The original data were downloaded from the DRYAD database (https://datadryad.org/stash). The dataset contained 716 samples and 127 observations. First, patients without CTD were removed; second, clinical observations with missing values greater than 5% were removed (**Supplementary Table 1**), and then the missing values were filled by multiple interpolations using the R package “mice”(v3.1.4) (19). We screened for clinical variables including general condition, comorbidities, biochemical indicators, and medication use. Finally, the samples were randomly divided into two parts, with 70% of the samples as the training cohort and 30% as the validation cohort.

### Screening of prognostic variables and construction of prognostic model

A univariate Cox regression analysis was used to screen for prognostic variables by using the R package “survival”(v3.5–0) (20). Prognostic variables were entered into the least absolute shrinkage and selection operator (Lasso) by using the R package “glmnet”(v4.1–3) (21). We used the “CV.glmnet” function (parameter default) for ten-fold cross-validation, and selected lambda.1se screened variables as the Lasso prognostic variables. Meanwhile, prognostic variables were also entered into a random survival forest (RSF) analysis to screen important prognostic variables based on the “rfsrc” function (parameters were set as mtry = 6, ntree = 1000) in R packet “randomForestSRC”(v3.1.0) (22). The top 10 variables with the greatest importance were selected as the prognostic variables of the RSF analysis. Overlapping prognostic variables were obtained by intersecting Lasso prognostic variables with RSF prognostic variables. The overlapping prognostic variables were entered into stepwise Cox regression (direction = both) to screen the hub prognostic variables, and then prognostic models were constructed based on the hub prognostic variables. The following risk score formula was obtained from the model (23):


$$Risk\;score=\sum_{i=1}^{n}Expi*\beta i$$

where *n, Expi*, and *βi* indicate the number of hub prognostic variables, variable values, and the stepwise Cox regression coefficient, respectively. In the training cohort, patients were divided into the high- and low-risk groups based on the median risk score, and the difference in prognosis between the two groups was assessed using the Kaplan–Meier analysis.

### Nomogram construction and performance evaluation

We constructed prognostic nomogram using the “nomogram” function (default parameters) by package “rms”(v6.2.0) (24). Calibration curves were plotted and the C-index was calculated to assess the predictive efficacy of the model. To assess the clinical benefit of the model, we plotted decision curve analysis (DCA) curves using the “dca” function in the R package “ggDCA”(v1.2) (25). In addition, to test the predictive power of the model in specific patients, we performed subgroup analyses of clinical variables (age, gender, diabetes mellitus, and interstitial lung disease). In the validation cohort, a Kaplan–Meier analysis, an evaluation of C-index, and the plotting of calibration curve were used to test the predictive power of the model.

### Statistical analysis

The study was statistically analyzed using R language (version 4.1.3). The mean ± standard deviation was used to represent continuous normally distributed variables, and the interquartile range was used to represent non-normally distributed information. A student’s *t*-test was used for the comparison of normally distributed random variables. The Wilcoxon rank test was used to compare non-normally distributed variables. In addition, categorical data were compared using the chi-square test or Fisher’s exact test. Stepwise Cox regression analyses and prognostic model constructions were applied using the R package “survival”. A survival analysis was performed using a Kaplan–Meier analysis and log-rank test.

## Results

### Baseline characteristics of pneumonia patients

Data for a total of 712 pneumonia patients treated with glucocorticoids, encompassing 127 variables, were downloaded from DRYAD. A total of 368 patients with CTD pneumonia were selected, and 84 variables had missing values of less than 5%. After multiple interpolations to supplement missing values, 37 variables were selected for analysis. These included age, gender, fever, cough, expectoration, chest pain, dyspnea, heart rate, respiratory rate, systolic pressure, diastolic pressure, cyanosis, CAP, bronchiectasia, interstitial lung disease, coronary heart disease, congestive heart failure, diabetes mellitus, anemia, white blood cells, neutrophils, lymphocyte, hemoglobin, platelets, aspartate aminotransferase, alanine aminotransferase, blood urea nitrogen, serum creatinine, K, Na, oxygen inhalation, high-dose glucocorticoid, immunosuppressant, ganciclovir, sulfanilamide, anti-aspergillus, and anti-pseudomonas. Patients were randomized into two parts, with 247 patients in the training cohort and 121 patients in the validation cohort. The groups were comparable at baseline except for the need for sulfanilamide treatment (**Table 1**).

**Table 1:** Clinical characteristics of patients with CTD pneumonia treated with glucocorticoids or/and immunosuppressants.

Characteristics	ALL (n=368)	Training set (n=247)	Validation set (n=121)	*P* value
Age >60 years, n (%)	194(52.7)	129(52.2)	65(53.7)	0.788
Gender, female, n (%)	228(62)	147(61.1)	81(63.6)	0.168
Symptoms and signs, n (%)				
Fever, n (%)	277(75.3)	185(74.9)	62(51.2)	0.813
Cough, n (%)	321(87.2)	217(87.9)	104(86.0)	0.607
Chest pain, n (%)	13(3.5)	9(3.6)	4(3.3)	1
Expectoration, n (%)	301(81.8)	201(81.4)	100(82.6)	0.767
Dyspnea, n (%)	215(58.4)	148(59.9)	67(55.4)	0.406
Laboratory examination				
White cell count, ×10^9^/L (IQR)	7.80(5.71, 11.18)	7.82(5.76, 11.08)	7.78(5.71, 11.41)	0.961
Neutrophils count, ×10^9^/L (IQR)	6.4(4.29, 9.80)	6.72(4.31, 10.02)	6.16(4.29, 9.01)	0.471
Lymphocyte count, ×10^9^/L (IQR)	0.83(0.5, 1.33)	0.81(0.48, 1.24)	0.84(0.54, 1.47)	0.357
Hemoglobin, g/L (IQR)	113(97.75, 128)	112(95.5, 128)	113(98, 128)	0.921
Platelet, ×10^9^/L (IQR)	184(127, 252.25)	184(128, 253)	184(127, 251)	0.871
Aspartate aminotransferase, U/L (IQR)	25(17, 42.25)	25(17.5, 43)	24(16, 38)	0.302
Alanine aminotransferase, U/L (IQR)	23(15, 44)	23(15, 43)	23(15, 44)	0.579
Blood urea nitrogen, mmol/L (IQR)	5.76(4.28, 8.15)	5.98(4.36, 8.11)	5.42(4.1, 8.35)	0.616
Serum creatinine, mmol/L (IQR)	59.95(48.9, 81.15)	59.9(49.2, 81.65)	61.6(47.3, 79.7)	0.862
K^+^, mmol/L (IQR)	3.9(3.6, 4.16)	3.9(3.6, 4.2)	3.8(3.5, 4.1)	0.289
Na^+^, mmol/L (IQR)	138(135, 140.9)	138(135, 140)	13.3(134, 141)	0.287
Persistent lymphocytopenia, n (%)	160(43.5)	111(44.9)	49(40.5)	0.419
Pneumonia severity index, n (%)	73(54, 96)	73(55, 96)	73(50, 97)	0.765
CURB-65 score >1, n (%)	105(28.5)	78(31.6)	27(22.3)	0.064
Underlying diseases				
Interstitial lung disease, n (%)	158(42.9)	144(58.3)	66(54.5)	0.494
Coronary heart disease, n (%)	47(12.8)	32(13.0)	15(12.4)	0.880
Congestive heart failure, n (%)	6(1.6)	3(1.2)	3(2.5)	0.399
Asthma, n (%)	6(1.6)	4(1.6)	2(1.7)	1
COPD, n (%)	44(12)	25(10.1)	19(15.7)	0.121
Bronchiectasia, n (%)	17(4.6)	9(3.6)	8(6.6)	0.203
Diabetes mellitus, n (%)	91(24.7)	62(25.1)	29(24.0)	0.813
tumor, n (%)	11(3)	6(2.4)	5(4.1)	0.352
Treatment, before admission				
Antibiotics, n (%)	241(65.5)	157(63.6)	84(69.4)	0.267
Antiviral drugs, n (%)	37(10.1)	25(10.1)	12(9.9)	0.951
Treatment, after admission				
High dose glucocorticoid, n (%)	140(38)	99(40.1)	41(33.9)	0.25
Ganciclovir, n (%)	152(41.3)	104(42.1)	48(39.7)	0.656
Sulfanilamide, n (%)	167(45.4)	124(50.2)	43(35.5)	0.008
Anti-aspergillus, n (%)	135(36.7)	87(35.2)	48(39.7)	0.406
Anti-pseudomonas, n (%)	282(76.6)	191(77.3)	91(75.2)	0.651
Complications				
Invasive mechanical ventilation, n (%)	89(24.2)	63(25.5)	26(21.5)	0.398
Non-invasive ventilation, n (%)	98(26.6)	66(26.7)	32(27.3)	0.955
Ventilation, n (%)	134(36.4)	91(36.8)	43(35.5)	0.807
Respiratory failure, n (%)	179(48.6)	123(49.8)	56(46.3)	0.526
ICU admission, n (%)	152(41.3)	106(42.9)	46(38.0)	0.37
ECMO, n (%)	15(4.1)	10(4.0)	5(4.1)	1
CAP, n (%)	337(91.6)	228(92.3)	109(90.1)	0.470

### Screening prognostic variables and constructing models

**Figure 2:**
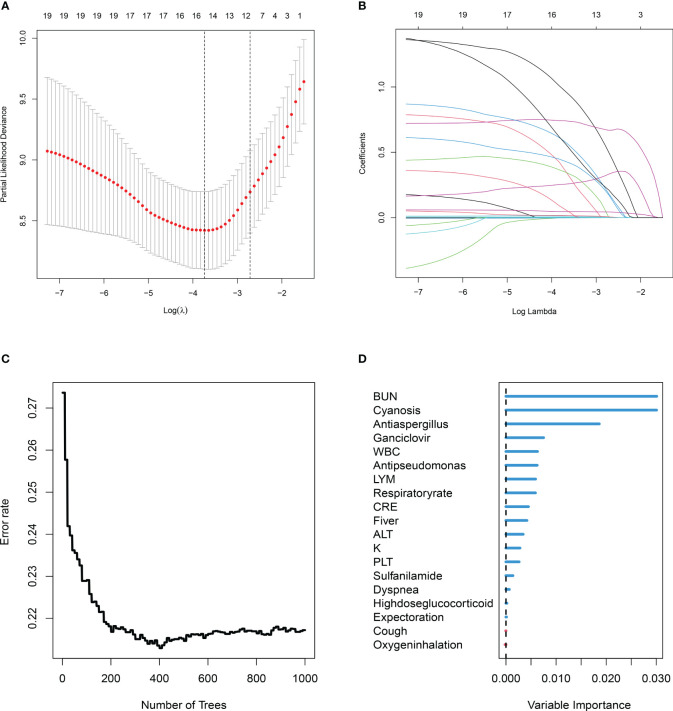
Two machine learning algorithms (least absolute shrinkage and selection operator (Lasso), random survival forest (RSF)) were used to screen prognostic variables. **(A)** Lasso coefficient profiles of the 19 clinic features, coefficient profile plot was produced against the log (λ) sequence. **(B)** Ten-fold cross-validation for the optimal parameter selection in Lasso. **(C)** RSF analysis of clinical variables. The x-axis represents the number of trees, and the y-axis represents the error rate. **(D)** 19 Variable importance was assessed by RSF. The *x*-axis represents variable importance and the *y*-axis represents clinical variables.

A univariate Cox regression analysis obtained 19 prognostic variables (**Table 2**). We included 19 prognostic variables in the Lasso Cox regression to reduce dimensionality. Ten-fold cross-validation was performed by the minimum criterion to the preferred parameters (**Figure 2A**). The trajectory of each prognostic index coefficient with the log-transformed lambda in the Lasso algorithm was observed (**Figure 2B**). We selected 11 variables corresponding to lambda.1se=0.066 as Lasso prognostic variables. Nineteen prognostic variables were also entered into the random prognostic forest (**Figures 2C, D**), which was eventually filtered to 10 significant prognostic variables as RSF prognostic variables based on variable importance scores. Eight overlapping prognostic variables (fever, respiratory rate, cyanosis, white blood cells, blood urea nitrogen, ganciclovir, anti-aspergillus, and anti-pseudomonas) were obtained by the two algorithms. The overlapping prognostic variables were put into a stepwise Cox regression analysis to obtain five variables (fever, cyanosis, blood urea nitrogen, ganciclovir, anti-pseudomonas), and a prognostic model was constructed based on these five variables. The model equation was risk score=1.389* Fever+0.990* Cyanosis +0.065* BUN +0.568* Ganciclovir +2.206*Anti-pseudomonas. The model coefficients and risk values for the fever variables can be seen in **Table 3**.

**Table 2:** Univariate Cox regression analysis obtained prognostic variables.

Variables	Univariate analysis	
	Hazard ratio (95% CI)	*P*
Fever	5.047 (2.030 to 12.551)	<0.001
Cough	5.237 (1.283 to 21.386)	0.021
Expectoration	2.619 (1.133 to 6.056)	0.024
Dyspnoea	3.653 (1.957 to 6.818)	<0.001
Respiratory rate	1.058 (1.028 to 1.089)	<0.001
Cyanosis	4.837 (2.990 to 7.824)	<0.001
Oxygen inhalation	3.182 (1.521 to 6.656)	0.002
White blood cells	1.076 (1.039 to 1.114)	<0.001
Lymphocyte	0.562 (0.358 to 0.881)	0.011
Platelets	0.997 (0.994 to 0.999)	0.015
Alanine aminotransferase	1.003 (1.002 to 1.005)	<0.001
Blood urea nitrogen	1.077 (1.053 to 1.102)	<0.001
Serum creatinine	1.002 (1.0001 to 1.004)	0.030
K	1.706 (1.121 to 2.596)	0.012
High-dose glucocorticoid	1.973 (1.224 to 3.181)	0.005
Ganciclovir	2.553 (1.564 to 4.166)	<0.001
Sulfanilamide	2.660 (1.578 to 4.485)	<0.001
Anti-aspergillus	4.089 (2.483 to 6.735)	<0.001
Anti-pseudomonas	23.768 (3.299 to 171.244)	0.001

**Table 3:** Variable risk coefficients and risk values.

Variables	Coefficient	HR	*P*
Fever	1.389	4.012 (1.558–10.331)	0.004
Cyanosis	0.990	2.690 (1.652–4.379)	<0.001
BUN	0.065	1.067 (1.040–1.094)	<0.001
Ganciclovir	0.568	1.765 (1.071–2.909)	0.026
Anti-pseudomonas	2.206	9.076 (1.229–67.031)	0.031

BUN, Blood urea nitrogen.

### Model evaluation

**Figure 3:**
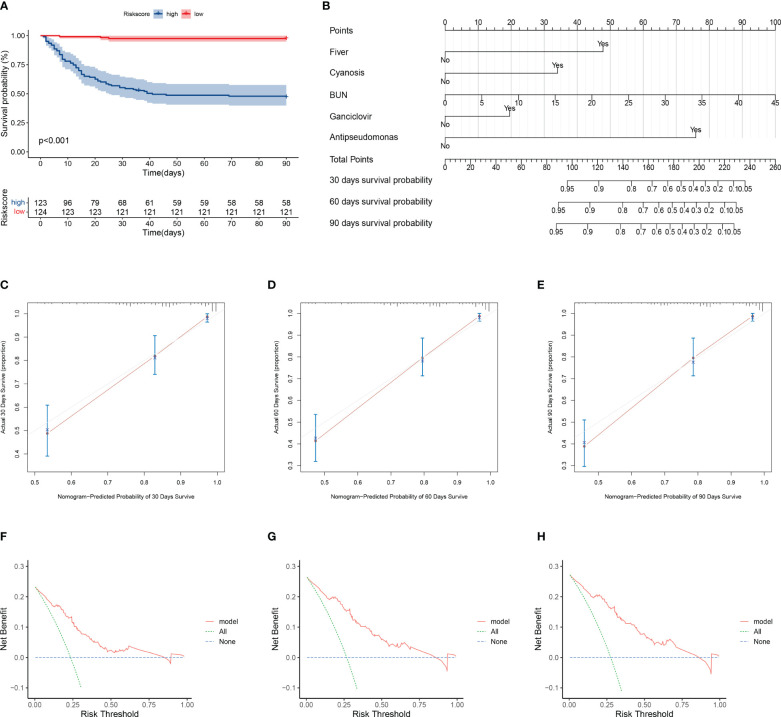
Evaluation of model prediction performance. **(A)** Kaplan–Meier analysis of prognosis for high- and low-risk groups. Red line indicates the low-risk group and green line indicates the high-risk group. **(B)** Nomogram prediction of 30-, 60-, and 90-day mortality in patients with pneumonia. Nomogram construction includes percent predicted values for fever, cyanosis, blood urea nitrogen, ganciclovir, and anti-pseudomonas incorporation. The corresponding values from each scale were then referenced to the predicted mortality at 30, 60, and 90 days. **(C–E)** Calibration curves of nomogram showing predicted 30-, 60-, and 90-day survival versus actual survival. **(F–H)** Decision curve analysis of the clinical benefit of model for predicting 30-, 60-, and 90-day mortality. The solid red line indicates the model, the blue dotted line indicates the hypothesis that no patients were treated, and the green dotted line indicates the hypothesis that all patients were treated.

**Figure 4:**
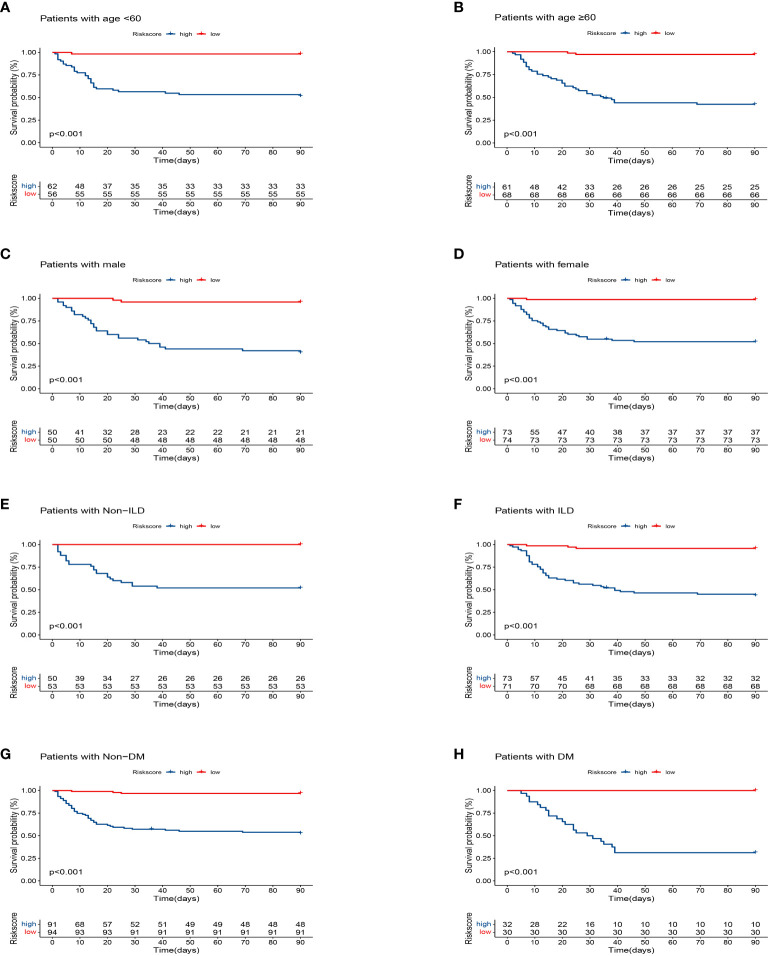
Kaplan–Meier analysis differences stratified by age (<60 and ≥60), gender (male and female), diabetes mellitus (DM) (with and non), interstitial lung disease (ILD) (with and non) between the high- and low-risk groups. **(A)** Age<60. **(B)** Age≥60. **(C)** Male. **(D)** Female. **(E)** Non-ILD. **(F)** ILD. **(G)** Non-DM. **(H)** DM.

The training cohort was included in the risk model and risk scores were calculated; patients were then divided into the high- and low-risk groups based on the median risk score, with significantly lower survival rates in the high-risk group than in the low-risk group (**Figure 3A**). Five independent variables were included in the nomogram to assess prognosis according to the results of the multivariable Cox regression analysis (**Figure 3B**). Predictive models performed well in predicting mortality: the C-index score was 0.808. Calibration curves for nomogram prediction of 30-day, 60-day, and 90-day overall survival were in good agreement with actual observations (**Figures 3C–E**). DCA results showed that the net benefit level of 30 days (risk threshold: 0~0.85), 60 days (risk threshold: 0~0.85), and 90 days (risk threshold: 0~0.85) predicted using the line chart was significantly higher than that of “no intervention” and “total intervention”, suggesting that the model had better clinical predictive efficacy (**Figures 3F–H**). In the subgroup analysis of age, gender, and comorbidities (interstitial lung disease, diabetes mellitus), survival time in the high-risk group was significantly lower than that in the low-risk group (*P<*0.001, **Figures 4A–H**). In conclusion, the above results indicate that our model has good predictive power.

### Model validation

**Figure 5:**
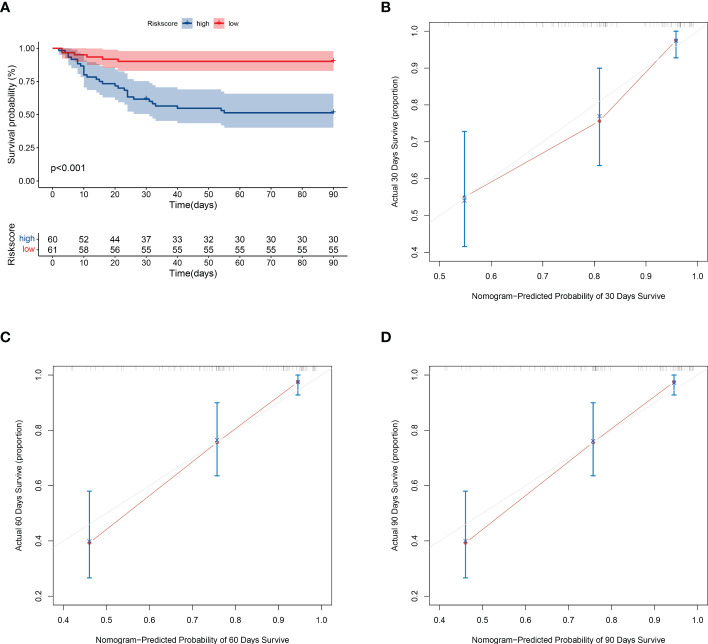
Validation of model prediction performance. **(A)** Kaplan-Meier analysis of prognosis in high- and low-risk groups. The red line indicates the low-risk group and the green line indicates the high-risk group. **(B–D)** Calibration plots of the model showing predicted 30-, 60-, and 90-day survival versus actual survival.

To verify the stability of the model, this study applied the model to the validation cohort. The high- and low-risk groups were divided according to the median value of the risk score, and the survival of the high-risk group was significantly lower than that of the low-risk group (**Figure 5A**). The prediction model performed well in predicting mortality: the C-index score was 0.762. The calibration plots of the model predicting overall survival at 30, 60, and 90 days agreed well with the actual observations (**Figures 5B–D**).

## Discussion

Immunosuppression-associated pulmonary infections are the most common site of CTD and may present with acute respiratory events (respiratory failure, ventilatory support) with a significant impact on mortality (26). This study used two machine learning algorithms to develop a model for predicting 90-day mortality in pneumonia patients with CTD that was being treated with glucocorticoids or/and immunosuppressants. The good predictive efficacy of the nomogram was also verified by the internal validation cohort. This model will be useful for predicting short-term survival in CTD pneumonia and could be used to intervene in risk factors at an early stage to improve prognosis.

We used two machine learning algorithms to obtain prognostic variables. Shiroshita et al. found low predictive performance when they used a single machine learning algorithm to construct a model for predicting in-hospital mortality in patients with chronic obstructive pulmonary disease pneumonia exacerbations (27). Ma et al. used multiple machine learning algorithms to construct a prognostic model for predicting the risk of death in patients with COVID-19 that was significantly superior to CURB-65 (28). This study used two algorithms to obtain models with few variables and good predictive value (C-index: 0.808, 0.762; training cohort, validation cohort, respectively).

A multivariable stepwise Cox regression analysis showed that five factors (fever, cyanosis, blood urea nitrogen, ganciclovir, and anti-pseudomonas) are prognostic risk factors for CTD pneumonia patients. Fever is a regulated increase in body temperature caused by the upward shift of the thermoregulatory point due to the action of a thermogenic source. Studies have reported that a longer duration of fever is a risk factor for poorer prognosis in children with severe adenovirus pneumonia (29). An analysis of clinical outcomes in COVID-19 pneumonia found that fever was a risk factor for poor prognosis (30). A study on determinants of outcome in children hospitalized with severe pneumonia found cyanosis to be a determinant of mechanical ventilation (31). Studies have reported that a high blood urea nitrogen/albumin ratio is a high-risk factor for long-term mortality in patients hospitalized with CAP (32). Some studies have reported a positive association between blood urea nitrogen and COVID-19 pneumonia severity and mortality (33). Another used machine learning algorithms (RSF, LASSO) to screen from more than 80 clinical variables to conclude that blood urea nitrogen was one of the predictors of mortality in patients with COVID-19 pneumonia and that patients with high blood urea nitrogen levels had higher mortality (34). Immunocompromised people are susceptible to severe macrophage pneumonia (35). The routine use of ganciclovir can alter the natural course of macrophage pneumonia infections (36). Some studies have also reported that specific antiviral therapies may improve clinical outcomes (37). However, our findings suggest that ganciclovir use is an independent risk factor for patients. A retrospective study of *Pseudomonas aeruginosa* bloodstream infections in children showed that *Pseudomonas aeruginosa* bloodstream infections occur mainly in immunocompromised patients and are associated with high mortality (38). Our study showed a poor prognosis for patients following the use of anti-pseudomonas.

In this study, a nomogram was created based on these independent risk factors. This study had some advantages over the PSI and the CURB-65. First, the model in this study did not include the age variable, which has stronger applicability than the PSI or the CURB-65. Second, the subjects of this study were patients with pneumonia using glucocorticoids or/and immunosuppressants for CTD. Compared with PSI and CURB-65, this model is suitable for specific populations. However, this study also has some limitations. First, this study was validated using an internal validation cohort and the results have not been further confirmed using an external validation cohort, and caution is needed in clinical use. Furthermore, this study was based on data collected from six hospitals in China, and the applicability of the model to Western cohorts needs further confirmation.

## Conclusions

This study developed a nomogram based on five variables to predict 90-day mortality in patients with CTD combined with pneumonia treated with glucocorticoids or/and immunosuppressants. The nomogram performed well in predicting 90-day mortality in patients with pneumonia.

## Data availability statement

The original contributions presented in the study are included in the article/**Supplementary Material**. Further inquiries can be directed to the corresponding authors.

## Ethics statement

Written informed consent was obtained from the individual(s) for the publication of any potentially identifiable images or data included in this article.

## Author contributions

Q-GL and JL conceived and designed the study. DL and LD performed the statistical analyses and wrote the manuscript text. Q-GL revised the manuscript. All authors contributed to and review the manuscript. All authors contributed to the article and approved the submitted version.
